# Impacts of Grazing Intensity and Plant Community Composition on Soil Bacterial Community Diversity in a Steppe Grassland

**DOI:** 10.1371/journal.pone.0159680

**Published:** 2016-07-28

**Authors:** Tong-bao Qu, Wei-chao Du, Xia Yuan, Zhi-ming Yang, Dong-bo Liu, De-li Wang, Li-jun Yu

**Affiliations:** 1 Institute of Grassland Science, Northeast Normal University, and Key Laboratory of Vegetation Ecology, Ministry of Education, Changchun, 130024, China; 2 College of Horticulture, Jilin Agricultural University, Changchun, 130118, China; 3 Dalian Medical University, Dalian, 116027, China; Shandong University, CHINA

## Abstract

Soil bacteria play a key role in the ecological and evolutionary responses of agricultural ecosystems. Domestic herbivore grazing is known to influence soil bacterial community. However, the effects of grazing and its major driving factors on soil bacterial community remain unknown for different plant community compositions under increasing grazing intensity. Thus, to investigate soil bacterial community diversity under five plant community compositions (Grass; *Leymus chinensis*; Forb; *L*. *chinensis* & Forb; and Legume), we performed a four-year field experiment with different grazing intensity treatments (no grazing; light grazing, 4 sheep·ha^−1^; and heavy grazing, 6 sheep·ha^−1^) in a grassland in China. Total DNA was obtained from soil samples collected from the plots in August, and polymerase chain reaction (PCR) analysis and denaturing gradient gel electrophoresis (DGGE) fingerprinting were used to investigate soil bacterial community. The results showed that light grazing significantly increased indices of soil bacterial community diversity for the Forb and Legume groups but not the Grass and *L*. *chinensis* groups. Heavy grazing significantly reduced these soil bacterial diversity indices, except for the Pielou evenness index in the Legume group. Further analyses revealed that the soil N/P ratio, electrical conductivity (EC), total nitrogen (TN) and pH were the major environmental factors affecting the soil bacterial community. Our study suggests that the soil bacterial community diversity was influenced by grazing intensity and plant community composition in a meadow steppe. The present study provides a baseline assessment of the soil bacterial community diversity in a temperate meadow steppe.

## Introduction

Soil biodiversity, a key determinant of the ecological and evolutionary responses of terrestrial ecosystems to current and future environmental change, has become a focus of soil ecological research field [1–2]. Ubiquitous soil bacteria possess enormous metabolic and physiological versatility and are essential to virtually all biogeochemical cycling processes [3]. Soil bacterial community is the basis for nutrient cycling, conversion and utilization, influenced by above- and belowground biota, which can have positive, negative or neutral impacts [4–6]. However, most previous studies have focused on indirect changes to soil bacteria caused by soil, plant and grazing, and scarce data are available on the interactions and mechanisms of aboveground herbivorous animals on bacterial community [7–9].

Soil bacterial communities may be regulated by the physicochemical characteristics of soil, such as texture, pH, water content, and nutrient concentrations (i.e., C, N, C/N), which are key determinants of soil bacterial growth and activity [10]. In general, bacterial diversity has a strong positive correlation with pH, moisture, soil organic carbon and nitrogen, and C/N ratios [11–12], though some experiments have also shown a negative correlation with pH [13]. Plant communities significantly alter microbial community composition through rooting patterns, rhizodeposition, water use, litter chemistry, and subsequent influences on soil properties and microclimate [14]. In recent decades, ecologists have begun to explore the relationships between belowground soil bacterial communities and their functional significance to plant communities and ecosystem processes [15]. The nutrients required by soil bacteria are frequently obtained from plant litter or through root exudates from living plants and root decay [16]. Greater plant production has the potential to lead to greater litter accumulation on the soil surface, and greater C inputs to soil result in greater soil organic C; these changes can have profound effects on soil microbial community composition [17–18].

Although most controlled experiments to date have focused on the responses of plant biomass and species, plant communities can also significantly alter soil bacterial community by changing soil physicochemical characteristics, such as pH, EC, and nutrient content [11, 19]. Additionally, plant root exudates influence the surrounding soil bacteria community, and certain plant species support a highly coevolved soil bacteria community [20]. Experiments have shown that changes in the plant diversity and composition of grassland ecosystems can lead to rapid responses in bacterial activity and diversity [21–22]. Furthermore, previous studies on terrestrial ecosystems have shown that the plant community composition tends to have a greater impact on soil microbial communities compared with plant species richness [19, 22–23]. Nonetheless, few consistent patterns have been detected between plant community composition and soil bacterial community.

The grazing of domestic herbivores significantly affects the vegetation and soil properties of grassland and thereby impacts soil bacterial communities through increased trampling, defoliation, and manure return [24–25]. Indeed, herbivores can alter soil functions by returning carbon and mineral nutrients as dung and urine deposition as well as by trampling, which often reduces soil aeration and moisture [3]. Such activities often modify C and N cycles, potentially changing C and N accumulation in the soil and impacting nitrogen and carbon cycling rates through changes in grassland plant communities [26–28]. Additionally, herbivores alter rhizosphere activity by increasing root exudation via the removal of biomass and effects on the litter breakdown dynamics in plant communities. Most studies have focused on grazing-induced changes in the plant species composition and soil properties of grassland mesocosms [29], whereas relatively few experimental studies have investigated how grazing combined with plant community composition affects soil bacterial community in meadow steppes [30]. Moreover, the associated ecological problems and interaction mechanisms are complex and require investigation through field trials and mesocosm experiments under different grazing intensities. The main objectives of this study were as follows: i. to investigate whether plant community composition affects soil bacterial community composition and diversity; ii. to explore how these changes are affected by intensity of grazing; and iii. to identify the main factors affecting the soil bacterial community in a meadow steppe.

## Materials and Methods

### Study site

The soil samples used in this study were obtained from an experimental grazing field located at Grassland Ecological Research Station of Northeast Normal University, Jilin Province, China (N 44°32´44"-58," E123°39´52"-40´17"; elevation 130–140 m). The climate is semiarid with a mean annual precipitation of 280–400 mm, with approximately 70% of the rainfall occurring from June to August. The annual mean temperature ranges from 4.6 to 6.4°C [31]. The grassland is a sodic saline meadow steppe with on average 35% clay, 45% silt, and 20% sand; the bulk density is 1.54 g·cm^-3^, and the average soil pH is approximately 8.7 [32]. The main vegetation type is meadow steppe dominated by the perennial grass *L*. *chinensis* (Trin.) Tzvel. Other companion species include *Phragmites australis* Trin., *Calamagrostis epigeios* Roth., *Chloris virgata* Swartz, *Lespedeza bicolor* Turcz., *Melilotus officinalis* (Linn.) Pall., *Medicago sativa* L., *Kalimeris integrifolia* Turcz., *Potentilla flagellaris* Willd. and *Carex duriuscula* C. A. M. [33–34].

### Experimental design and animal management

Based on previous research, we first collected evidence for the relationships between aboveground plant community composition and belowground bacterial community diversity to determine whether the correlations are a result of direct associations among the groups of organisms above and below the surface.

For this experiment, 10 ha of grassland that had been mowed for several decades was fenced in 2009 to exclude large herbivores (S1 Fig). For four years, an experiment of different grazing intensities (no grazing: CK; light grazing: LG; heavy grazing: HG) and different plant community composition (Grass; *Leymus chinensis*; Forb; *L*. *chinensis* & Forb; and legume) was performed in a meadow steppe in northeastern China. Three grazing intensities were arranged in a randomized complete block design with three replications, and the stocking rates were 0, 4, and 6 sheep·ha^−1^, respectively [35]. We analyzed plant diversity in the nine blocks, with a total of 112 plant species. All sampled plant community plots were divided into five compositions: Grass (perennial rhizosphere grass mixture of *Phragmites australis*, *Calamagrostis epigeios*, and *Chloris virgata*); *L*. *chinensis* (a clonal dominant species and it played an important role in structuring the plant community); Forb (perennial forb mixture of *Kalimeris integrifolia*, *Potentilla flagellaris*, and *Carex duriuscula*), *L*. *chinensis* & Forb (mixture of *L*. *chinensis* and perennial forb at a composition of 33 or 67%); and Legume (mixture of *Lespedeza daurica*, *Melilotus officinalis*, and *Medicago sativa*). From 2009 to 2012, the grazing period was approximately 5 months per year from May to September. Grazing occurred twice a day from 6:00 am to 8:00 am and from 4:00 pm to 6:00 pm [36].

### Soil sampling

Soil samples were collected from 225 plots (five plant community compositions, five replicates in each nine blocks) in mid-August 2011. Five sampling replicates were obtained from the upper 5–20 cm (d = 2.5 cm) using a soil auger; these five cores (0.4 kg fresh weight) were bulked together and divided into two sub-samples after sieving (2 mm^2^ mesh) to remove coarse roots and stones. All of the samples were stored at 4°C prior to transport to the laboratory. One sub-sample was stored at 4°C for physicochemical analysis, and the other was stored at -20°C for denaturing gradient gel electrophoresis (DGGE) analysis.

### Physicochemical analysis

The soil samples (100–200 g wet weight) were air dried and then passed through a 0.14 mm sieve. The soil pH and EC were determined using a soil-water ratio of 1:5. The soil water content (SW) before air drying was obtained by the oven-drying method. The soil organic carbon (SOC) content was measured using the Mebius method and by Walkley-Black acid digestion. The TN content was determined using an autoanalyzer (Foss 2100, FOSS Kjeltec^®^) with the Kjeldahl method following vitriol digestion. The total phosphorus (TP) content was measured colorimetrically after P extraction by Na_2_CO_3_ fusion.

### Total DNA extraction from soil samples and DGGE analysis

Total DNA was obtained directly from each soil sample by CTAB-based extraction using a described protocol [37]. In brief, 500 mg of soil was mixed with 250 mg of 0.1 and 0.5 mm (1: 1) zirconia–silica beads, 500 mL of phenol–chloroform–isoamyl alcohol (25: 24: 1; Tris saturated, pH 8.0) and 500 mL of extraction buffer (12.2 mM KH_2_PO_4_, 112.8 mM K_2_HPO_4_, 5% w/v CTAB, 0.35 M NaCl; pH 8.0). The soil samples were bead-beaten for 30 s at 50 ms^-1^ and then centrifuged at 10 000 x g for 5 min at 4°C. The supernatant was mixed with 500 mL of chloroform–isoamyl alcohol (24: 1) and centrifuged again at 10 000 x g for 5 min at 4°C. The supernatant was precipitated at room temperature for 2 h with two volumes of a 30% w/v PEG 6000 and 1.6 M NaCl solution. The precipitated nucleic acids were pelleted by centrifugation at 10 000 x g for 10 min at 4°C.

The amount of DNA obtained from the samples varied from 10 to 20 ng·μl^−1^. The bacterial communities were then assessed by PCR amplification of cDNA templates using the general bacterial primers 16S 341F-GC and 518R. Each reaction mixture contained 50 ng DNA, 1 U Taq DNA Polymerase (Invitrogen^®^), 1.5 mM MgCl_2_, 0.2 mM each dNTP, 5 pmol each primer, and 1 μL DMSO to a final volume of 25 μL. The amplification reactions started with an initial denaturation step at 95°C for 5 min, followed by 30 cycles of 30 s at 95°C, 30 s at 55°C and 1 min at 72°C, and a final extension at 72°C for 10 min. Approximately equal amounts of the PCR products were loaded onto 6% (w/v) polyacrylamide gels prepared with 0.5× Tris-acetate-ethylene diamine tetraacetic acid buffer and denaturing gradients ranging from 45% to 65%. Electrophoresis was performed for 16 h at 100 V and 60°C [37–38].

The DGGE gels were scanned using a GS-800 Imaging Densitometer (Bio-Rad), and the bacterial community fingerprints of the DGGE bands were analyzed using Quantity One software (version 4.4.1; Bio-Rad). The images were normalized using markers, and cluster analysis was performed by applying the unweighted pair-group method with mathematical averages (UPGMA) using the similarity matrix generated was based on the Dice coefficient.

### Statistical analysis

One-way analysis of variance (ANOVA) and Turkey’s multiple comparison tests were applied for post-hoc analysis of the significant differences among the factors. Two-way ANOVAs were then performed. Soil bacterial diversity indices were calculated, (a) Shannon-Wiener index(*H*): *H* = -Σ(*Pi*×ln*Pi*), was calculated based on gel band intensity; (b) Richness (*R*): *R* = *S*, was calculated based on converting gel image into binary; (c) Pielou evenness index (*E*): *E*_*H*_ = *H/H*_*max*_ = *H*/ln*S*; Pi = Ni /N, where Ni is the height of the peak and N the sum of all peak heights in the densitometric curve, the significance level was set at *P* < 0.05, all of the data analyses were performed with SAS (Statistical Analysis Software). To evaluate relationships between microbial community composition and environmental variables, we first ordinated the DGGE presence/absence data using nonmetric multidimensional scaling (NMDS) and then looked for significant correlations between the ordination axes and environmental variables, and the statistical differences between soil bacterial fingerprints were measured using ANOSIM (analysis of similarity). The NMDS ordination and ANOSIM test were implemented by R software Version 3.1.1.

## Results

### Changes in soil properties along a grazing gradient with different plant community compositions

**Fig 1:**
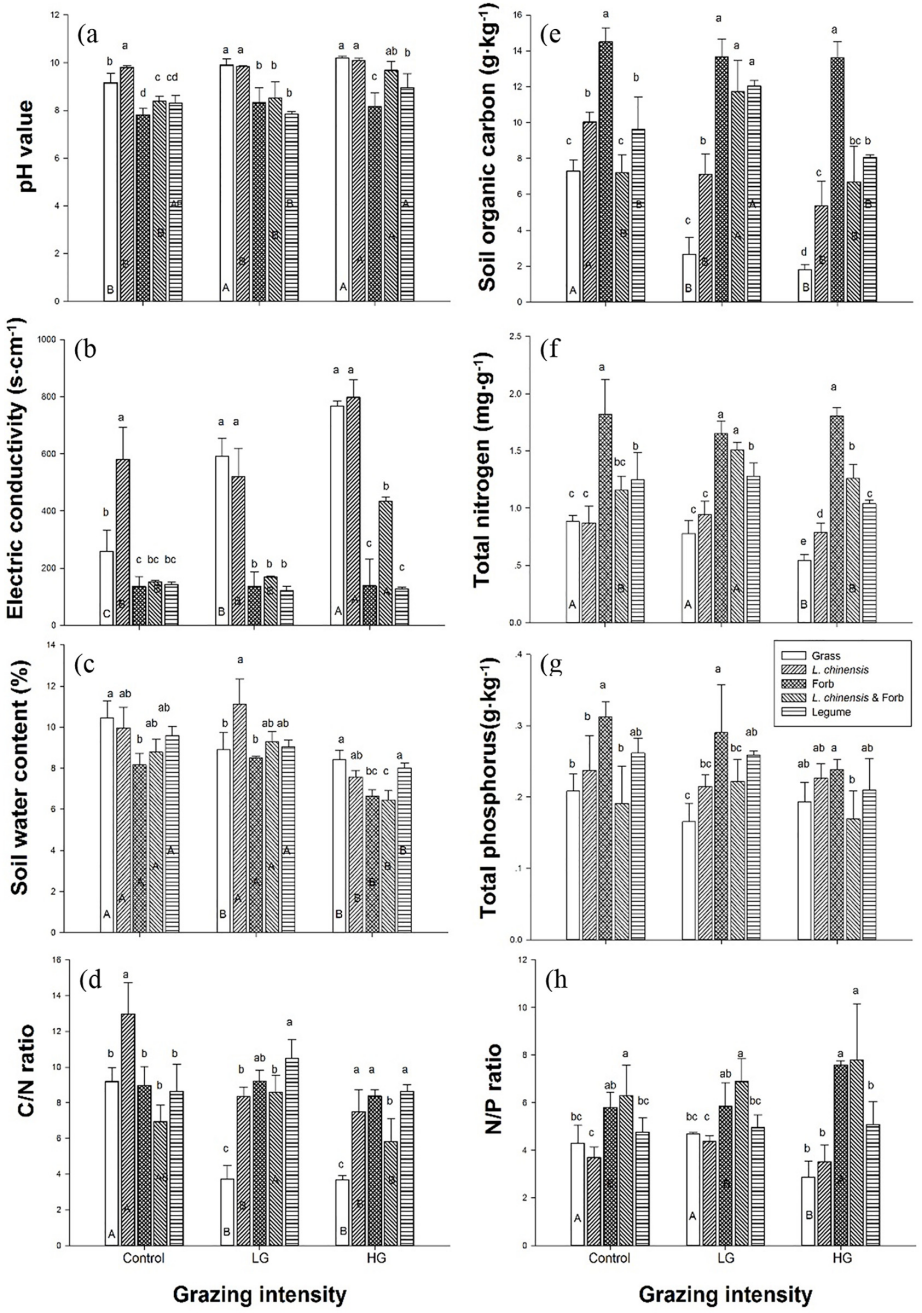
Soil property responses to different plant community composition along a grazing gradient. (a) pH value, (b) electrical conductivity, (c) soil water content, (d) the C/N ratio, (e) soil organic carbon, (f) total nitrogen, (g) total phosphorus and (h) the N/P ratio. Values represent the means ± SE. Significant differences between different plant community composition levels within a grazing intensity are indicated by lowercase letters (a-c); significant differences between different grazing intensities within a plant community composition level are indicated by capital letters (A-C; *P* ≤ 0.05). *Vertical bars* ± 1 SE.

ANOVA detected large and significant differences among the different plant community compositions for all of the soil physicochemical properties along the grazing gradient (Fig 1, Table 1). In general, grazing enhanced the pH value in the Grass, *L*. *chinensis*, *L*. *chinensis* & Forb, and Legume groups but not in the Forb group (*P* < 0.001), especially under heavy grazing (Fig 1a). The soil pH was significantly higher in the Grass and *L*. *chinensis* groups (*P* < 0.001). The results for EC were similar to those of pH under the grazing treatments (*P* < 0.001): EC in the Grass group increased along the grazing gradient, whereas high EC values in the *L*. *chinensis* and *L*. *chinensis* & Forb groups were only observed with heavy grazing (*P* < 0.001) (Fig 1b). Grazing reduced SW in the Grass group, but only heavy grazing reduced this parameter in the other plant community compositions (*P* < 0.001) (Fig 1c). Comparatively, SW was significantly higher in the *L*. *chinensis* group and lower in the Forb group in light grazing (*P* < 0.001) (Fig 1c).

**Table 1:** Two-way analysis of variance of the effects of grazing and plant community composition on soil pH, electrical conductivity (EC), soil water (SW), soil organic carbon (SOC), total N (TN), total P (TP), C/N and N/P ratios.

Factors		Grazing intensity (G)	Plant functional group (P)	G × P
d.f.		2,45	4,45	8,45
pH	*F*	14.164	40.484	2.499
	*P*	<0.001	<0.001	0.033
EC	*F*	48.285	149.463	13.734
	*P*	<0.001	<0.001	<0.001
SW	*F*	49.122	12.641	3.636
	*P*	<0.001	<0.001	0.005
SOC	*F*	24.862	96.275	9.620
	*P*	<0.001	<0.001	<0.001
TN	*F*	4.737	80.301	2.747
	*P*	0.016	<0.001	0.021
TP	*F*	3.857	10.567	1.280
	*P*	0.032	<0.001	0.291
C/N	*F*	24.094	26.352	10.450
	*P*	<0.001	<0.001	<0.001
N/P	*F*	0.905	21.313	2.201
	*P*	0.415	<0.001	0.056

The grazing intensity treatments and plant community compositions significantly affected SOC (*P* < 0.001), TN (*P* < 0.001), and TP (*P* < 0.001) (Fig 1e, 1f and 1g). Grazing decreased SOC in the Grass and *L*. *chinensis* groups (*P* < 0.001), whereas light grazing increased SOC in the *L*. *chinensis* & Forb and Legume groups (Fig 1e). Heavy grazing decreased TN in the Grass group, and light grazing increased it in the *L*. *chinensis* & Forb group. The SOC, TN and TP contents were highest in the Forb group and lowest in the Grass group. Overall, grazing reduced the C/N ratio (*P* = 0.016) (Fig 1d) but not the N/P ratio (*P* = 0.415) (Fig 1h).

### PCR-DGGE analysis of soil bacterial communities

**Fig 2:**
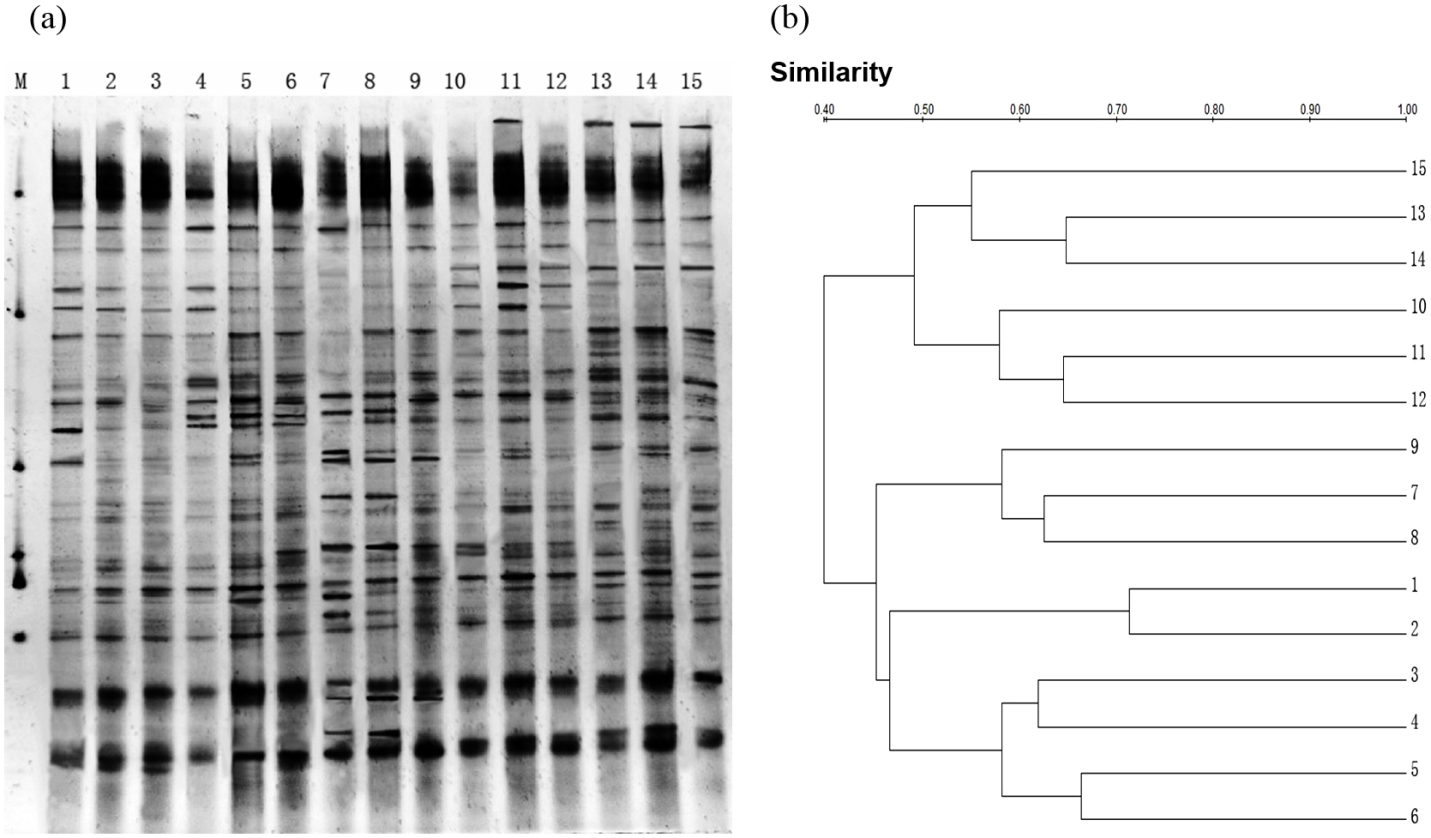
PCR-DGGE analysis of soil bacterial 16S rDNA (a) and dendrogram constructed using the soil bacterial community fingerprints (b) along the grazing gradient with different plant community compositions. M: marker, *L*. *chinensis*: 1–3, Grass: 4–6, Legume: 7–9, *L*. *chinensis* & Forb: 10–12, Forb: 13–15. Control: 1, 4, 7, 10, 13; light grazing: 2, 5, 8, 11, 14; and heavy grazing: 3, 6, 9, 12, 15. Difference between the profiles are indicated by the similarity percentage. The dendrogram of the cluster analysis was based on the Dice coefficient and UPGMA clustering algorithm.

PCR-DGGE analysis of the extracts yielded virtually identical profiles for the fifteen samples analyzed (Fig 2). The diversity of the soil bacterial community was affected by both grazing intensity and plant community composition. The samples were characterized by the presence of a limited number (11–19) of strong bands and a larger number of weak bands; most of the differences were observed in both the strong and weak bands. In Fig 2a, more bands are observed in lanes 1, 4, 8, 11, 13 and 14, indicating more complex bacterial communities in the Grass, *L*. *chinensis* and Forb groups under no grazing and in the Grass, Legume, *L*. *chinensis* & Forb and Forb groups under light grazing. Conversely, few bands are present in lanes 3, 6 and 12, indicating that heavy grazing decreased the complexity of the bacterial communities in the *L*. *chinensis*, Grass and *L*. *chinensis* & Forb groups (Fig 2a).

UPGMA cluster analysis (Dice coefficient of similarity) of the DGGE patterns confirmed these general observations (Fig 2b). All of the lanes clustered together at a level of approximately 39% similarity. The following lanes were clustered together: lanes 13, 14 and 15; lanes 10, 11 and 12; lanes 7, 8 and 9; lanes 1 and 2; and lanes 3, 4, 5 and 6. The bacterial communities under *L*. *chinensis* and Grass were more evidently clustered together. Additionally, a clear trend was observed, in which the same plant community composition resulted in bacterial communities presenting 49% similarity with the other bacterial communities.

### Effects of grazing intensity and plant community composition on bacterial diversity indices

**Fig 3:**
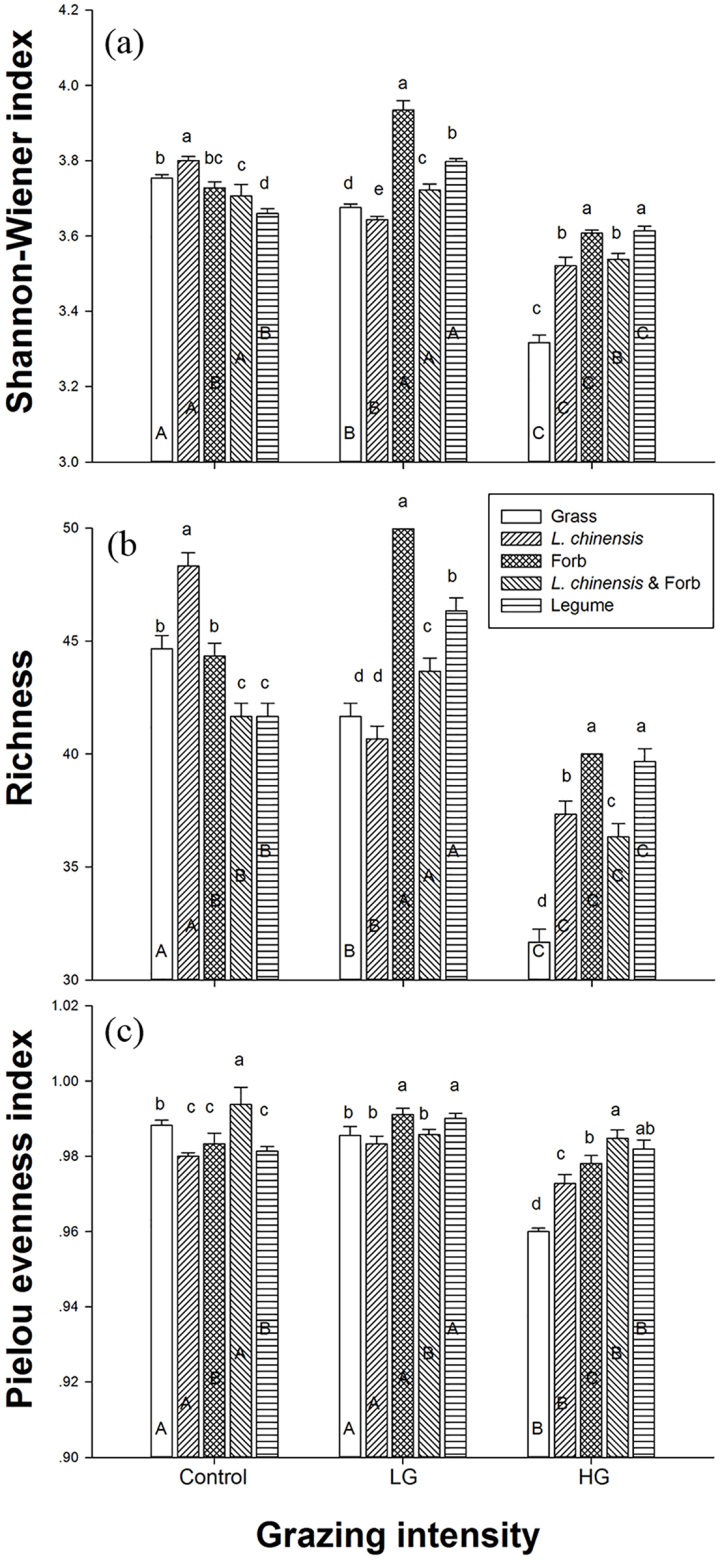
Effects of grazing on bacterial diversity. (a) Shannon-Wiener index, (b) richness, and (c) Pielou evenness index. Error bars denote the SE. Significant differences between different plant community composition levels within a grazing intensity are indicated by lowercase letters (a-c); significant differences between different grazing intensities within a plant community composition level are indicated by capital letters (A-C; *P* ≤ 0.05). *Vertical bars* ± 1 SE.

The grazing intensity and plant community composition significantly affected bacterial diversity indices. A significant decrease in the Shannon-Wiener index was observed with different grazing intensities under Grass and *L*. *chinensis*, especially with heavy grazing. Light grazing significantly increased the Shannon-Wiener index under Forb and Legume, whereas heavy grazing significantly decreased this index. Only heavy grazing significantly decreased the Shannon-Wiener index under *L*. *chinensis* & Forb (Fig 3a). The result for richness was similar to that for the Shannon-Wiener index (Fig 3b) under the different grazing intensities. The Shannon-Wiener index (*P* < 0.001) and richness (*P* < 0.001) were significantly higher under *L*. *chinensis* and lower under *L*. *chinensis* & Forb and Legume in the control treatment but were significantly higher under Forb and lower under Grass in the light grazing and heavy grazing treatments (Table 2) (Fig 3a and 3b).

**Table 2:** Two-way analysis of variance of the effects of grazing intensity (G) and plant community composition (P) on the Shannon-Wiener index, richness and Pielou evenness index of the soil bacterial community.

Factors		Grazing intensity (G)	Plant functional group (P)	G × P
d.f.		2,45	4,45	8,45
Shannon-Wiener index	*F*	179.326	209.240	258.555
	*P*	<0.001	<0.001	0.033
Richness	*F*	821.438	150.406	106.906
	*P*	<0.001	<0.001	<0.001
Pielou evenness index	*F*	123.511	34.515	26.110
	*P*	<0.001	<0.001	<0.001

Heavy grazing significantly decreased the Pielou evenness index under Grass and *L*. *chinensis* compared with the control and light grazing treatments. Light grazing significantly increased the Pielou evenness index under Forb, but heavy grazing significantly decreased this index. In addition, grazing decreased the Pielou evenness index under *L*. *chinensis* & Forb, though significant changes were not observed between light grazing and heavy grazing. Light grazing significantly increased the Pielou evenness index under Legume (Fig 3c). The heavy grazing and control treatments significantly increased the Pielou evenness index (*P* < 0.001) under *L*. *chinensis* & Forb, though light grazing produced higher index values under Forb and Legume (Table 2) (Fig 3c).

### Effects of grazing intensity and plant community composition on the soil bacterial community composition

**Fig 4:**
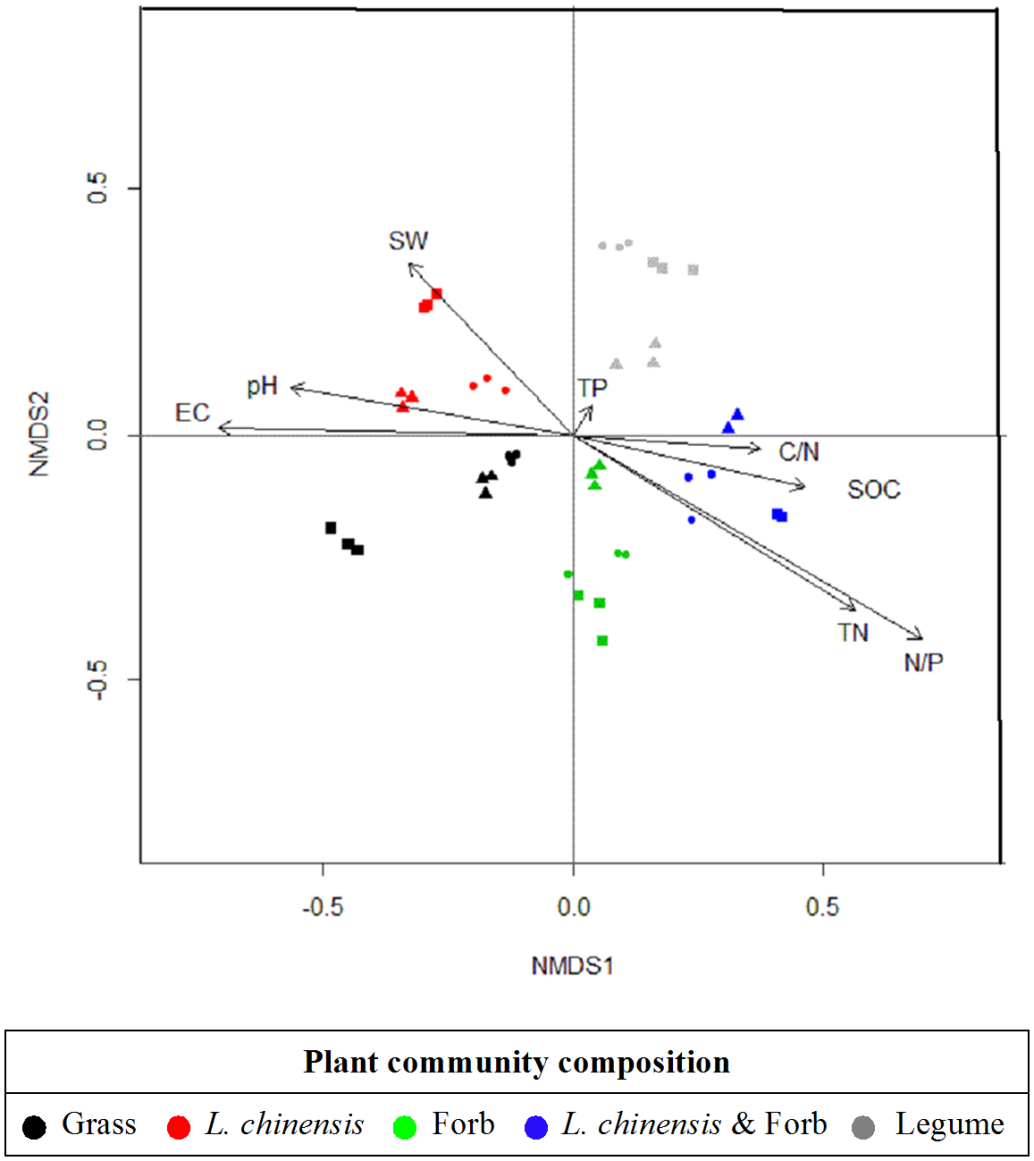
NMDS ordination of the DGGE profiles of 16S rRNA gene fragments with superimposed vectors derived from soil chemistry data. Circles refer to the control, triangles refer to light grazing and squares refer to heavy grazing. The statistical differences between soil bacterial fingerprints were measured using ANOSIM (analysis of similarity).

NMDS revealed the effects of grazing intensity and plant community composition on the soil bacterial community composition (*P* < 0.001), closer points imply more similar soil bacterial community compositons, Stress = 0.16 for all ordinations, confirming a good correlation between the data and its ordination (Fig 4). Bacterial community compositons differ significantly between the five plant community composition (ANOSIM test, *P* = 0.001), no significantly between the three grazing intensities (ANOSIM test, *P* = 0.851), which indicates that plant community composition is the key factor that triggers the variation of soil bacterial community composition.

Among all of the soil properties examined, the NMDS plot revealed that the variables explaining the most important factors governing bacterial community composition were N/P ratio (*r*^*2*^ = 0.657, *P* = 0.001), EC (*r*^*2*^ = 0.503, *P* = 0.001), and TN (*r*^*2*^ = 0.444, *P* = 0.001), the other factors were pH (*r*^*2*^ = 0.330, *P* = 0.002), SW (*r*^*2*^ = 0.231, *P* = 0.004), SOC (*r*^*2*^ = 0.224, *P* = 0.006), and C/N ratio (*r*^*2*^ = 0.142, *P* = 0.053) (S1 Table).

## Discussion

The objective of this work was to determine the influence of plant community composition on the diversity of soil microbial communities under grazing intensity treatments and to examine whether these effects are context dependent. Our findings showed that grazing intensity and plant community composition altered the soil bacterial community, and the soil N/P ratio, electrical conductivity (EC), total nitrogen (TN) and pH were the major environmental factors affecting the soil bacterial community. These results provide evidence that partly supports the previously reported changes in ecological processes caused by large herbivore grazing.

### Effects of grazing intensity and plant community composition on soil bacterial community diversity

Grazing livestock can play an important role in the microbial ecology of grasslands through a series of specific factors, including plant community composition and biomass, fecal and urine deposition, rhizosphere exudation, and soil texture and physicochemical properties; these factors can have positive, neutral or negative effects on soil bacterial diversity [7,15,39–41]. Our study clearly showed that the diversity of the soil bacterial community varied significantly along the gradient from light to heavy grazing intensity (Fig 3; Table 2). This result is consistent with that of previous studies in which light grazing was shown to markedly increase but heavy grazing to significantly decrease the Shannon-Wiener index, richness, and Pielou evenness index of the soil bacterial community from an upland grassland and an Inner Mongolian dry steppe [42–44]. Previous studies and our results clearly indicate that changes in soil bacterial community diversity are closely related to different grazing intensities [4, 7, 44]. In the present study, light grazing had a limited effect on the pH, EC, SWC and TN of the soil, whereas heavy grazing significantly decreased the parameters of SWC, SOC, TN and the C/N ratio (Fig 1).

Some experiments demonstrated that light or moderate grazing can optimize the aboveground net primary productivity via compensatory growth, which may have occurred due to an increase in nutrient availability (C, N or others), thereby facilitating vegetation regrowth [45–46]. In contrast, heavy grazing may decrease the storage availability of soil C and N and result in a reduction in soil bacterial diversity [47]. Soil pH and moisture are considered to be important factors that affect microbial community composition and diversity, and the soils in the studied grassland are mixed saline and alkaline (pH 8.0–10.0). Other studies have suggested that bacterial diversity declines as the soil pH increases from neutral to alkaline [48]. Indeed, compared with the no grazing treatment, heavy grazing significantly increased pH (from 9.1 to 10.2 under Grass) and EC (from 258.7 to 767.7 under Grass) but decreased SW (from 10.5 to 8.4% under Grass) (Fig 1; Table 1); these changes jointly constrain soil bacterial diversity in nutrient-poor soils [8, 49].

Soil bacteria are mostly heterotrophic and utilize plant exudates or decomposing plant material for growth. Although the reduction in food quantity and changes in food quality caused by alterations to the plant community structure or composition often modify the abundance, activity, and diversity of soil bacterial communities, few studies have focused on it [19, 21, 50]. We found that the diversity of the soil bacterial community differed markedly under the studied plant community composition in the meadow steppe environment. Our results showed that the soil bacterial diversity indices were higher under Grass and *L*. *chinensis* than under Forb and Legume in the no grazing plots (Fig 3). Alterations in the plant community composition often result in different structural and chemical compositions of the plant litter that is returned to the soil, subsequently causing different depletion patterns of soil resources [51]. The meadow steppe in our study site is dominated by the perennial grass *L*. *chinensis* and other companion grasses, such as *P*. *australis*, *C*. *epigejos* and *C*. *virgata*, a plant composition that is most likely to produce greater litter decomposition and soil exudate profiles under the *L*. *chinensis* and Grass groups. Consequently, the organic acid in the root exudates may have reduced the stress of soil salinity and pH and increased soil bacterial diversity at the study site.

Furthermore, the changes in soil bacterial diversity could have been caused by interactions between grazing intensity and plant community composition (Table 2; Fig 3). Our results showed that light grazing significantly increased soil bacterial diversity under the Forb and Legume groups but markedly decreased diversity under the *L*. *chinensis* and Grass groups (Fig 3). Previous experiments have shown that light and moderate grazing could lead to overcompensation due to the amounts of litter and root exudates in grassland ecosystems [31, 52–54]. Light grazing increased plant productivity by increasing the quantity of plant litter under the Forb and Legume groups (Fig 3), which may have promoted soil C and N dynamics by accelerating root production and exudation. These conditions are known to favor bacterial growth in soils [55]. However, heavy grazing decreased the Shannon-Wiener index, phylotype richness, and Pielou evenness index of the soil bacterial diversity under all of the plant community compositions (Fig 3). As the forage utilization rate increased, the amount of plant litter returning to the soil decreased with increases in grazing intensity [46]. In particular, heavy grazing led to decreased soil quality, fertility and moisture but promoted the soil pH and EC, which might have decreased the soil bacterial diversity in each plot (Figs 1 and 3). Experiments have also documented the effects of heavy grazing on aboveground plant communities and belowground microbial communities [3–4, 7, 46].

In addition, we observed the highest soil bacterial diversity under Forb and the lowest under Grass with grazing (Fig 3). Forb is composed of a variety of species (perennial forb mixture of *Kalimeris integrifolia*, *Potentilla flagellaris*, and *Carex duriuscula*), and heavy grazing altered the composition of Forb, adding some new species that produce more kinds of root exudates and plant secondary metabolites. This led to the highest SOC, TN, and TP values and the lowest pH under Forb, whereas heavy grazing reduced the Grass biomass, with no change in species composition. Different plant community compositions may create different niche dimensions by varying the litter quantity and quality, consequently inducing changes in groups of soil decomposers [19]. Moreover, trampling by grazers altered the soil texture and physicochemical properties, which could affect soil bacterial community.

### Main factors affecting soil bacterial community

Although the results of our study showed that the grazing intensity and plant community composition influenced the soil bacterial community diversity (Fig 3), the latter also significantly affected the soil bacterial community composition (Fig 4), NMDS of the DGGE data revealed that soil N/P ratio (*r*^*2*^ = 0.657), EC (*r*^*2*^ = 0.503), TN (*r*^*2*^ = 0.444), and pH (*r*^*2*^ = 0.330) were the major factors driving soil bacterial community (Fig 4). Previous work has shown that soil bacterial communities are primarily affected by factors such as soil pH, moisture, organic C and N, and aboveground plant species, community composition and diversity in grassland soils [3, 56–59]. In addition, experimental evidence has suggested that soil moisture is a key driving factor in dry steppe and semi-arid steppe ecosystems [60–61], and soil organic C or the C/N ratio plays an important role in controlling bacterial community composition and diversity in alkaline permafrost-affected soils [62–63]. However, these results are not consistent with our observations. The nutrient levels in the studied meadow steppe were low (TN 0.872–1.823 mg·g^-1^) and precipitation high (280–400 mm), and the soil bacterial diversity of this site was mainly influenced by soil pH and EC, consistent with studies in extreme and/or moderate saline environments [64–66].

Near-neutral soils present a greater availability of nutrients to support copiotrophic bacteria, whereas high pH and EC conditions significantly reduce utilization of soil nutrients in alkaline-saline regions [67]. In general, near-neutral pH levels might be regarded as a proxy for the physiological availability of a variety of nutrients. The internal pH of bacterial cells is normally close to neutral, and an external pH environment similar to this intracellular value may suggest a reduction in the energy expenditure required to maintain this internal pH and fewer specialized adaptations [63, 68]. However, high pH could be considered a “stressful” environment because of grazing-induced disturbances and changes in plant community traits; such environments might require specialized adaptations that relatively few taxa have been able to acquire [67]. At our study site, heavy grazing significantly increased pH (from 9.1 to 10.2) and EC (from 258.7 to 767.7) and decreased TN (from 0.88 mg·g^-1^ to 0.55 mg·g^-1^), thereby resulting in a reduction in soil bacterial diversity (Shannon-Wiener index, richness and Pielou evenness index) (Figs 1 and 3). Thus, it is clear that multiple stresses due to herbivore grazing lead to additive negative effects on soil bacteria diversity through changes in soil physical and chemical properties in meadow steppe ecosystems.

## Conclusions

The experimental results presented here suggest that grazing intensity and plant community composition as well as their interactions markedly influence the Shannon-Wiener index, phylotype richness, and Pielou evenness index of the soil bacterial diversity. Light grazing significantly increased the diversity of the soil bacterial community under the Forb and Legume groups but notably decreased diversity under the *L*. *chinensis* and Grass groups. In contrast, heavy grazing greatly decreased the diversity of the soil bacterial community diversity for all of the plant community compositions. Our study suggests that the soil bacterial community diversity is influenced by grazing intensity and plant community composition, soil N/P, EC, TN, and pH to be the main driving forces affecting soil bacterial community in this meadow steppe at a regional scale. The results of this study reveal that the diversity of the soil bacterial community is influenced by grazing intensity, plant community composition and soil physicochemical properties. The present study provides a baseline assessment of the soil bacterial community in a temperate meadow steppe and could be a useful tool for future assessments of the effects of grassland management.

## Supporting Information

S1 Fig Schematic map of the location on grazing intensity site. (TIF)

S1 Table Fits of soil property vectors onto NMDS ordinations. (DOC)
