# Supplementary figures and images for: Impacts of Grazing Intensity and Plant Community Composition on Soil Bacterial Community Diversity in a Steppe Grassland
Source: PLoS One. 2016 Jul 28;11(7):e0159680. doi: 10.1371/journal.pone.0159680 (PMC4965099; doi:10.1371/journal.pone.0159680)

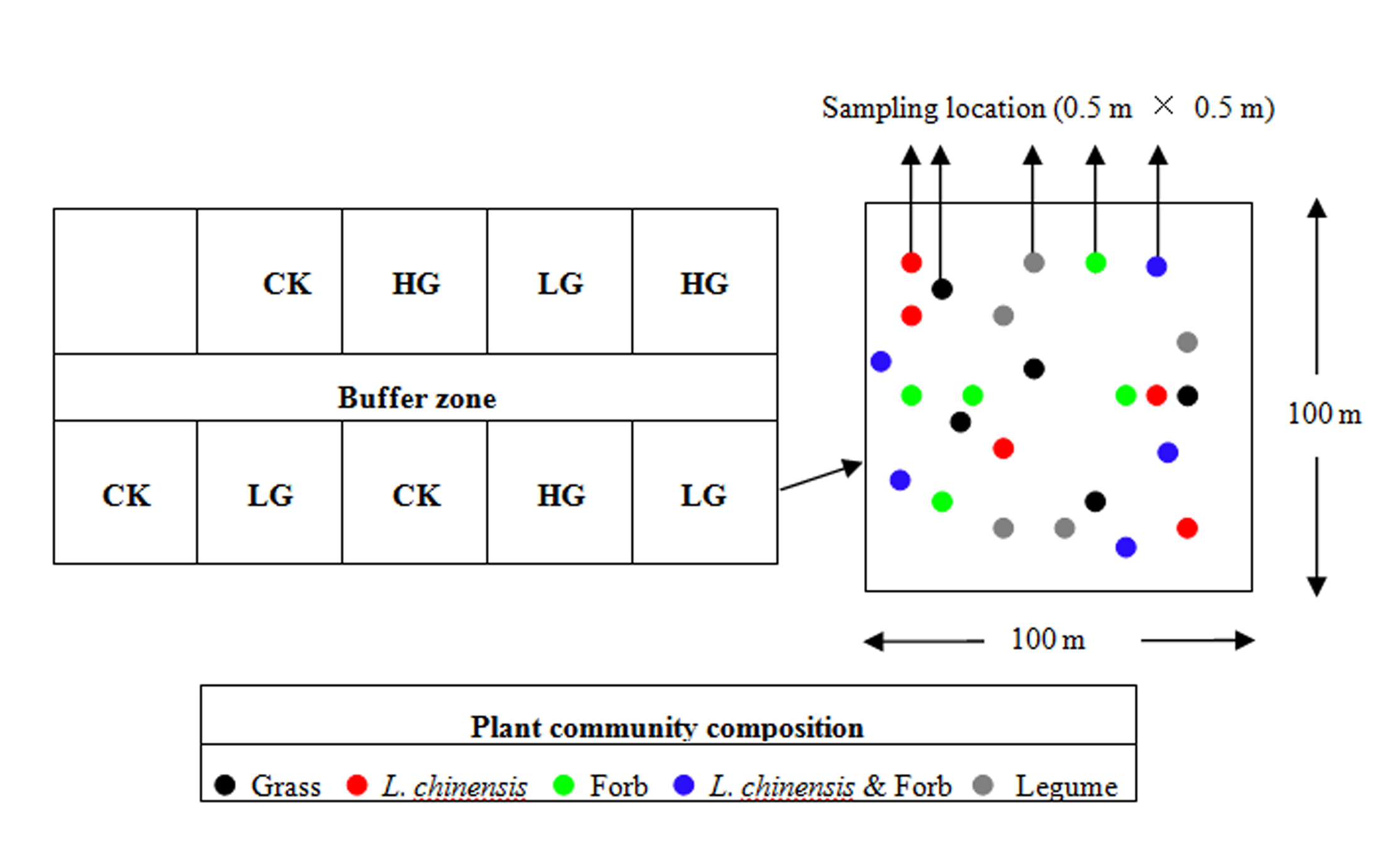

Supplement: S1 Fig — (TIF) [file pone.0159680.s001.tif]
