# Supplementary material for: Impacts of Grazing Intensity and Plant Community Composition on Soil Bacterial Community Diversity in a Steppe Grassland
Source: PLoS One. 2016 Jul 28;11(7):e0159680. doi: 10.1371/journal.pone.0159680 (PMC4965099; doi:10.1371/journal.pone.0159680)
Supplement: S1 Table — (DOC) [file pone.0159680.s002.doc]

**S1 Table** **Fits of soil property vectors onto NMDS ordinations.**

|  | NMDS1 | NMDS2 | *r^2^* | *P* |
| --- | --- | --- | --- | --- |
| C | -0.45350 | -0.89126 | 0.0114 | 0.765 |
| pH | -0.98584 | 0.16771 | 0.3301 | 0.002 |
| EC | -0.99978 | 0.02094 | 0.5028 | 0.001 |
| SW | -0.68608 | 0.72753 | 0.2313 | 0.004 |
| SOC | 0.97424 | -0.22549 | 0.2244 | 0.006 |
| TN | 0.84439 | -0.53572 | 0.4440 | 0.001 |
| TP | 0.51792 | 0.85543 | 0.0050 | 0.895 |
| C**/**N | 0.99731 | -0.07335 | 0.1418 | 0.053 |
| N**/**P | 0.85889 | -0.51217 | 0.6573 | 0.001 |
